# Inclusion of Magnesium- and Strontium-Enriched Bioactive Glass into Electrospun PCL Scaffolds for Tissue Regeneration

**DOI:** 10.3390/polym17111555

**Published:** 2025-06-03

**Authors:** Francesco Gerardo Mecca, Nathália Oderich Muniz, Devis Bellucci, Cécile Legallais, Timothée Baudequin, Valeria Cannillo

**Affiliations:** 1 Dipartimento di Ingegneria “Enzo Ferrari”, Università degli Studi di Modena e Reggio Emilia, 41125 Modena, MO, Italy; francescogerardo.mecca@unimore.it (F.G.M.); valeria.cannillo@unimore.it (V.C.); 2 Centre National de la Recherche Scientifique (CNRS), Biomechanics and Bioengineering (BMBI), Centre de Recherche Royallieu, Université de Technologie de Compiègne, 60200 Compiègne, France; natclio@hotmail.com (N.O.M.); cecile.legallais@utc.fr (C.L.); timothee.baudequin@utc.fr (T.B.)

**Keywords:** bioactive glass, PCL, electrospinning, biological assay, ion doped, mechanical properties

## Abstract

Bioactive glass (BG) is a promising material known for its osteogenic, osteoinductive, antimicrobial, and angiogenic properties. For this reason, melt-quench-derived BG powders embedded into composite electrospun poly(ε-caprolactone) (PCL) mats represent an interesting option for the fabrication of bioactive scaffolds. However, incorporating BG into nano-/micro-fibers remains challenging. Our research focused on integrating two BG compositions into the mat structure: 45S5 and 45S5_MS (the former being a well-known, commercially available BG composition, and the latter a magnesium- and strontium-enriched composition based on 45S5). Both BG types were added at concentrations of 10 wt.% and 20 wt.%. A careful grinding process enabled effective dispersion of BG into a PCL solution, resulting in fibers ranging from 500 nm to 2 µm in diameter. The mats’ mechanical properties were not hindered by the inclusion of BG powder within the fibrous structure. Furthermore, our results indicate that BG powders were successfully incorporated into the scaffolds, not only preserving their properties but potentially enhancing their biological performance compared to unloaded PCL electrospun scaffolds. Our findings indicate proper cell differentiation and proliferation, supporting the potential of these devices for tissue regeneration applications.

## 1. Introduction

In recent years, research into regenerative medicine has focused on identifying new techniques for manufacturing supports for tissue regeneration, commonly referred to as scaffolds. Among these, electrospinning has gained significant attention from the research community due to its wide range of applications, ease of setup, and cost-effectiveness [1,2]. This technique enables the fabrication of nano- or micro-fibrous, porous, unwoven flat scaffolds (or mats) by applying a high electrical potential difference between a needle containing a polymer dissolved in solvents and a collector [3]. Research into soft and bone tissue engineering began exploring this technique as a viable alternative for fabricating scaffolds with nano- to micro-scale structures, as it proves particularly useful in soft tissue regeneration due to its ability to mimic the architecture of the extracellular matrix [4]. One of the strengths of electrospinning is the possibility of producing fibers with a tunable morphology in the 10^−9^–10^−6^ m size range [1]. This enables the fabrication of scaffolds with a high surface-area-to-volume ratio, similar to the extracellular matrix. These unique characteristics make electrospun fibers ideal candidates for tissue regeneration [5]. However, the high tunability of the mats’ properties also requires an optimization of the machine parameters. Even minimal changes in the polymeric solution, machine parameters, or environmental conditions can lead to significantly different results in the final product [6].

The incorporation of bioactive glass particles (specifically in the nano- to micro-size range) into the fiber structure may expand the range of applications of both BG and electrospun mats. BGs are a category of biomaterials known to be resorbable, non-toxic, and bioactive [7]. Furthermore, BGs are known for a range of properties, including promoting osteogenesis, exhibiting antibacterial effects, and facilitating specific body responses, such as angiogenesis [8]. Among the various BGs created over the years, 45S5 Bioglass^®^ (commonly referred to as 45S5, composition [7]: 46.1 mol% SiO_2_, 26.9 mol% CaO, 24.4 mol% Na_2_O, 2.6 mol% P_2_O_5_) was the first BG to be developed and the first to be approved for in vivo use [7]. For this reason, it is often used as reference material in literature. Another BG composition, 45S5_MS (composition [9]: 46.1 mol% SiO_2_, 2.6 mol% P_2_O_5_, 9.4 mol% Na_2_O, 26.9 mol% CaO, 5 mol% MgO, 10 mol% SrO) was recently developed and tested for its biological properties by Bellucci et al., showing very promising results, specifically for applications in the field of soft tissue engineering [9]. The 45S5_MS is a composition derived from 45S5, in which strontium and magnesium were introduced to partially substitute for calcium as a modifier. The addition of these ions into the BG composition is motivated by their positive biological effects, which are widely reported in the literature [10,11,12].

The inclusion of BG nano- to micro-sized particles into the structure of electrospun mats has attracted significant interest due to its potential applications in tissue engineering. Specifically, electrospun mats for regenerative medicine containing BG may serve as viable alternatives for functionalizing these composite scaffolds [5,13,14,15]. However, the use of powdered melt-derived BGs presents a major hurdle when suspended in the electrospinning solution. In fact, achieving a homogeneous dispersion of the BG particles can be challenging, as an agglomeration of BG particles and physical alterations of the fiber morphology may occur.

Poly(ε-caprolactone) is an FDA-approved semicrystalline polymer that has been extensively studied in biomedical applications due to its excellent mechanical properties and adjustable biodegradability [16,17]. PCL-based electrospun fibers have been widely used in several applications, including bone substitutes, cranial implants, and cardiac patches, among others [18,19,20,21,22]. Furthermore, recent studies have demonstrated that scaffolds composed of PCL and BG offer advantages over other PCL/ceramic composites, such as those based on β-tricalcium phosphate or hydroxyapatite, due to their superior mechanical properties and faster degradation kinetics, making them more suitable for bone tissue engineering [23].

Numerous previous works have shown that BGs, when in contact with a biological environment, tend to react, leading to the precipitation of hydroxyapatite (HA), one of the main mineral constituents of bone tissue—a phenomenon known as bioactivity [7,24]. Over the past two decades, specific tests, such as simulated body fluid (SBF) testing, have consistently proven to be an effective method to determine whether a biomaterial, such as BG, can precipitate HA when exposed to a biological environment [25]. Even considering the limitations of this test, its role is still considered pivotal for characterizing BG-containing devices. The interaction between PCL and SBF has been reported to induce non-trivial effects on polymers, such as swelling, weight loss, and acidification of the SBF solution [26,27,28], a phenomenon that contrasts with the typical alkalizing behavior of BGs related to ion leaching [29].

For these reasons, considering the potential alkalinization of the solution [30] and potential ion release (specifically of magnesium in 45S5_MS [31]), in vitro biological investigations can provide valuable complementary information on the overall performance of the scaffolds, offering insights into their potential for in vivo studies [25].

The aim of this research was to investigate the feasibility of electrospinning a PCL/BG suspension containing melt-derived BG, taking into account the challenges of achieving a homogeneous dispersion of BG particles within the electrospun mat, as well as the more frequent use of sol–gel-derived compositions [32,33,34,35]. Furthermore, we investigated whether the incorporation of BG powder into the structure of electrospun mats could influence the biological response of these devices, particularly in terms of cell differentiation and proliferation, in order to test previous evidence supporting the potential application of 45S5_MS in soft tissue engineering.

## 2. Materials and Methods

### 2.1. Materials

#### 2.1.1. Materials for PCL/BG Electrospun Mats

PCL beads (MW = 80 kDa), dichloromethane (DCM), and dimethylformamide (DMF) were purchased from Sigma Aldrich Chimie S.a.r.l (Saint-Quentin-Fallavier, France). Raw oxides and carbonates (SiO_2_, Ca_3_(PO_4_)_2_, CaCO_3_, NaCO_3_, MgCO_3_, and SrCO_3_) used for the preparation of the BGs were acquired from Carlo Erba Reagents S.r.L. (Rodano-Milano, Italy).

#### 2.1.2. Materials for Biological Studies

Mouse fibroblast-like cells (L929, ATCC), immortalized adipose-derived stem cells (ASC52telo, ATCC), mesenchymal stem cell basal medium (ATCC-PCS-500-030), mesenchymal stem cell growth kit-low serum for adipose, and umbilical cord-derived MSCs (ATCC-PCS-500-040) were purchased from LGC Standards (Teddington, UK). BCIP/NBT alkaline phosphatase (ALP) substrate solution, DAPI, dexamethasone (D8893), β-glycerophosphate disodium salt hydrate (G9422), and L-ascorbic acid (A4544) were purchased from Sigma Aldrich Chimie S.a.r.l (Saint-Quentin-Fallavier, France). Dulbecco’s Modified Eagle’s Medium—low glucose, Osteocalcin (OCN) polyclonal antibody, goat anti-Rabbit IgG (H+L) cross-adsorbed secondary antibody Alexa Fluor™ 488, penicillin and streptomycin (PenStrep), and L-glutamine were purchased from ThermoFisher Scientifics (Eindhoven, The Netherlands). Dulbecco’s phosphate-buffered saline (DPBS) and bovine serum albumin (BSA) were obtained from Dominique Dutscher SAS (Bernolsheim, France). Fetal bovine serum (FBS) was provided by Cytiva (Marlborough, MA, USA).

### 2.2. Methods

#### 2.2.1. Bioactive Glass Preparation

Both 45S5 and 45S5_MS were prepared via a traditional melt–quench technique [7,9]. Raw oxides and carbonates were weighed and subsequently mixed in a rotary laboratory mixer for 4 h. The powder mixture was then melted in a platinum crucible using a furnace (Nannetti S.r.L., Faenza, Italy). The following thermal cycle was applied: heating from room temperature to 1100 °C at a rate of 10 °C/min, followed by an isothermal hold at 1100 °C for 1 h. A second heating phase was then performed, increasing the temperature from 1100 °C to 1450 °C at 10 °C/min, followed by another isothermal hold at 1450 °C to ensure complete melting of the powders. The molten glass was subsequently quenched in room-temperature water to obtain a frit, which was then dried at 110 °C for 12 h in an oven. Detailed information on the composition of 45S5 and 45S5_MS is presented in Table 1.

The glass frit was milled into a fine BG powder. To obtain micro- to submicrometric particle sizes, a two-step milling process was used. First, the glass frit was ground using alumina milling balls and a jar inside a laboratory mill MGS1800/2 (MGS S.r.l., Fiorano Modenese, Italy) for 20 min. The ground BG was then sieved using a 63 µm sieve.

A second, more thorough, grinding step was performed using a PM100 (Retsch Gmbh, Haan, Germany) to obtain a finer powder. Ten grams of BG powder were placed inside a steel-reinforced zirconia jar with 5 mm-diameter zirconia milling balls. The jar was spun at 600 rpm for 20 min. This operation was repeated 3 times, for a total duration of 1 h. This milling process was carried out in three steps to maximize efficiency while allowing heat dissipation.

Both BGs underwent this procedure to obtain two final powders. Finally, to ensure that the BGs were thoroughly milled, a scanning electron microscope (SEM) (Quanta FEG 250, FEI, Hillsboro, OR, USA) was used to qualitatively analyze their morphology.

#### 2.2.2. Electrospinning of PCL/BG Scaffolds

As previously discussed, electrospinning is an advanced scaffold fabrication technique used to manufacture flat polymeric mats composed of thin, micro- to nanometric fibers entangled together [1,6]. Electrospun mats were obtained from a 12 wt.% precursor solution of PCL and BG powder. First, the BG powder was dispersed in 60 vol% DCM and 40 vol% DMF. DCM acts as the solvent for PCL, while DMF is usually added as a stabilizer of the electrospinning jet, because of its higher dielectric constant and lower vapor pressure [36]. The ratio 3:2 DCM/DMF was chosen based on previous experiments. Subsequently, the appropriate amount of PCL beads was added to the BG/solvent mixture. The resulting suspension was stirred for 4 h. Four different suspensions were produced: (1) 90% of PCL and 10% of BG, (2) 80% of PCL and 20% of BG, each prepared using either 45S5 or 45S5_MS glass. Table 2 presents a summary of the compositions of each sample.

The electrospinning parameters needed extensive optimization to achieve stable and efficient spinning of the suspension. A comprehensive overview of these parameters is presented in Table 3. Previous work by Baudequin et al. [22] was used as a reference for the manufacturing of these scaffolds. Briefly, the voltage was maintained between 17.8 kV and 20.0 kV to balance the quantity of suspension drawn out of the needle [37]. The working distance was fixed at 15 cm, and the flow rate was set to 1.5 mL/min. The suspension was fed through a 19-gauge, 8 cm long needle, chosen specifically to prevent clogging caused by BG agglomerates. A rotating cylinder collector was used at a speed of 300 rpm.

The surfaces of the electrospun mats were studied using a field-emission scanning electron microscope (FE-SEM—Fei Nova NanoSEM 450, ThermoFisher Scientific, Eindhoven, The Netherlands), coupled with an energy-dispersive X-ray spectroscopy (EDS) detector QUANTAX-200 (Bruker Corp., Billerica, MA, USA). EDS mapping was performed to detect the presence of silicon in the scaffolds, serving as an indicator of BG within the electrospun mats. Small sections of the samples were cut and sputter-coated with a 10 nm gold layer. ImageJ (an open-source image analysis software—version 1.54j, NIH, Bethesda, MD, USA) was used to determine the mean fiber diameter. To achieve this, at least 50 measurements were taken. A one-way ANOVA statistical analysis was performed to detect the statistical variance between the results. Moreover, a qualitative analysis based on visual observation to detect the BG particles was conducted to confirm their presence in the electrospun scaffolds.

The electrospun mats underwent a thermal gravimetric analysis (TGA) using an STA 429 CD (Netzsch GmbH, Selb, Germany) to quantitatively analyze the presence of BG powder in the electrospun mats as residue after thermal degradation of the PCL. Samples weighing 10 to 20 mg were heated at a rate of 20 °C/min to 600 °C. This temperature was chosen to avoid any possible crystallization occurring in the BG structure [9].

#### 2.2.3. Mechanical Characterization

The mechanical properties of the electrospun mats were evaluated through uniaxial tensile testing using a traction test machine, Synergie 400 (MTS System Corp., Eden Prairie, MN, USA), equipped with a 100 N load cell and clamps for direct measurement. A tensile speed of 1 mm/min at room temperature was used to measure the samples’ properties. The partial alignment of the fibers was considered a potential factor influencing the results. For this reason, two types of samples were cut from each mat: one along the longitudinal (L) direction, and one along the transversal (T) direction. The L-direction is parallel to the radial component of the cylinder’s velocity, while the T-direction is parallel to the rotation axis of the collector cylinder. Five 5 × 20 mm strips were cut for each type of sample. The clamps of the traction test machine were set at a starting distance of 5 mm. The thickness of the samples was measured using a digital feeler gauge. Finally, the Young’s modulus (E) and ultimate tensile strength (σ_MAX_) were calculated from the obtained tensile stress–strain curve. A two-way ANOVA statistical analysis was conducted to evaluate the variance of the results.

#### 2.2.4. Surface Wettability

The hydrophilicity of the samples, a key parameter for understanding their ability to support cell adhesion on their surfaces, was measured [38]. The water contact angles of the mats were assessed at room temperature using an automatic optical contact angle system (OCA 15EC, Dataphysics Instruments GmbH, Stuttgart, Germany) equipped with a 6.5× zoom lens. A 20× magnification was used to capture the measurements. Distilled water was dropped onto the surface of each sample and immediately photographed (t = 0) to measure the water contact angle. Each sample was measured at least 10 times at different locations to obtain an average value. A one-way ANOVA test was performed to assess the differences in the contact angles among the scaffolds. A significance level of α = 0.05 was considered for all statistical evaluations.

#### 2.2.5. Methods for Tests in Simulated Body Fluid

Bioactivity tests were carried out according to Kokubo’s method. The SBF solution was prepared in the lab by slowly and carefully mixing the salts under constant stirring. HCl was used to buffer the solution and adjust its pH to 7.4. The required quantity of SBF was calculated using the formula provided by Kokubo et al. [25]:

(1) $${V\;}_{SBF}=\frac{S_{A}}{10}$$

where *V_SBF_* is the volume of SBF in which the samples are soaked, and *S_A_* is the total surface area of the sample.

The electrospun mats were cut into 45 × 5 mm strips and weighed using an analytical balance. The strips were then secured in Petri dishes, submerged in SBF, and incubated at a constant temperature of 37 °C. Every 48 h, the pH of the solution was measured before replacing it with fresh, unreacted SBF. The samples were extracted at three predetermined time points: 3, 7, and 14 days. After the immersion period, the samples were removed from the SBF, washed with demineralized water, and dried at 40 °C for at least 3 days to remove the residual solution from the pores. The scaffolds were then analyzed using an environmental scanning electron microscope (Fei Quanta 200, ThermoFisher Scientific, Waltham, MA, USA) equipped with an EDS detector (Inca, Oxford Instruments, Oxford, Abingdon, UK).

#### 2.2.6. Biological Evaluation

##### Indirect Cell Culture

The cytotoxicity of the scaffolds was assessed in accordance with ISO 10993-5 guidelines [39] to evaluate the presence of cytotoxic compounds released into the media. The viability of the mouse fibroblast-like cells (L929) in indirect contact with the scaffolds was evaluated.

The cells were cultured and maintained in flasks with complete DMEM, supplemented with 10% FBS, 1% penicillin–streptomycin, and 1% L-glutamine, at 37 °C in a humidified atmosphere containing 5% CO_2_. For the test, 96-well plates containing 100 µL of complete DMEM were seeded with 10,000 cells/well and incubated for 24 h. In parallel, electrospun mats were cut into small pieces, covered with complete DMEM, and incubated under agitation for 24 h at 37 °C and 5% CO_2_. Before the test, the mats were disinfected by immersion in 70% EtOH for 30 min and then washed three times with DPBS under sterile conditions. Latex was used as a negative control (CTRL (−)) and it was disinfected using the same procedure.

After 24 h, the culture medium was replaced with 100 µL of the mat- or latex-extracted medium and incubated for another 24 h in the same conditions (*n* = 3). Complete DMEM served as the positive control (CTRL (+)).

Following incubation, 20 µL of the MTS solution were added to each well and incubated for 2 h at 37 °C and 5% CO_2_. The optical density (OD) was measured at 492 nm using a microplate reader (Tecan Spark 10, Männedorf, canton of Zürich, Switzerland). The absorbance values were normalized to the positive control and expressed as relative cell viability (%).

##### Direct Cell Culture

In vitro cell tests were performed using human immortalized adipose-derived stem cells (ASC52telo). To assess the effect of BG presence, a pure PCL electrospun mat was used as a control.

Cells were cultured in complete mesenchymal stem cell basal medium, supplemented with 10% FBS, 1% penicillin–streptomycin, and 1% L-glutamine, at 37 °C and 5% CO_2_. The electrospun mats were disinfected by immersion in 70% EtOH for 30 min and washed three times with DPBS under sterile conditions. They were then incubated overnight on a 24-well plate in complete medium at 37 °C and 5% CO_2_. The mats were seeded with ASC52telo cells at a density of 10,000 cells per sample (Ø = 12 mm).

To evaluate the influence of the scaffolds on cell activity, two groups were established: one cultured in complete medium (proliferation group) and the other in osteogenic medium (complete medium supplemented with 1 M β-glycerophosphate, 50 mg/mL L-ascorbic acid, and 100 µM dexamethasone). The culture was maintained for 28 days, with medium changes every two days.

##### Qualitative Staining of Alkaline Phosphatase

After 28 days of culture, the medium from both groups was removed, and 500 µL of BCIP/NBT alkaline phosphatase substrate were added to each well. The samples were incubated in the dark at room temperature for 30 min. The substrate solution was then removed to stop the reaction, and the samples were washed with DPBS.

##### Immunofluorescence (IF) Staining of Osteogenic Bone Marker

The presence of osteogenic markers in the in vitro cultured samples was analyzed by IF-OCN staining. Samples cultured with ASC52telo for 14 and 28 days were collected, washed with phosphate-buffered saline (PBS), and fixed in 4% paraformaldehyde for 30 min. They were then permeabilized with 0.5% Triton X-100 for 20 min, washed three times with PBS, and saturated with 2% BSA for 2 h. The samples were incubated overnight at 4 °C with the primary OCN antibody. After washing with PBS and 0.1% BSA, the samples were incubated for 1 h at room temperature with the goat anti-rabbit IgG (H+L) Cross-adsorbed secondary antibody Alexa Fluor™ 488 and DAPI in a humid chamber. The samples were visualized using a confocal laser scanning microscope (LSM 980 AiryScan II, Carl Zeiss France S.A.S., Rueil-Malmaison, France), with excitation/emission wavelengths of 405 nm for DAPI (blue) and 488 nm for OCN (green).

## 3. Results and Discussion

### 3.1. Characterization of the Electrospun Scaffolds

**Figure 1:**
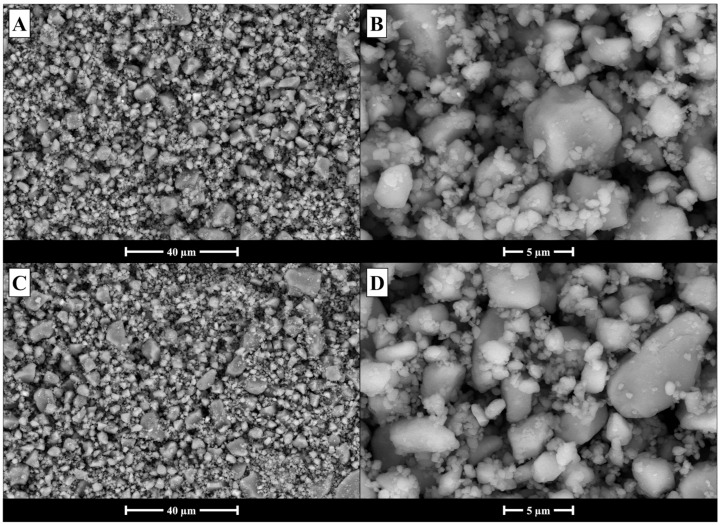
SEM analysis of the morphology of the powdered BG: (**A**,**B**) 45S5; (**C**,**D**) 45S5_MS samples, after milling with the PM100 rotary mill.

As previously described, the BG powders underwent an extensive milling process to ensure optimal results during suspension spinning. To evaluate the morphology of the powders after milling, SEM imaging was performed. The results are shown in Figure 1. The BG appears to have been successfully ground into a powder with dense particles and variable geometry. However, a significant agglomeration of the powder was observed, with smaller BG particles adhering to one another. This phenomenon, typical of nano-sized BG particles, has been previously reported in the literature and is attributed to residual surface charges [40,41,42]. The use of cyto-compatible deflocculants may mitigate this effect by improving particle dispersion within the suspension [42].

**Figure 2:**
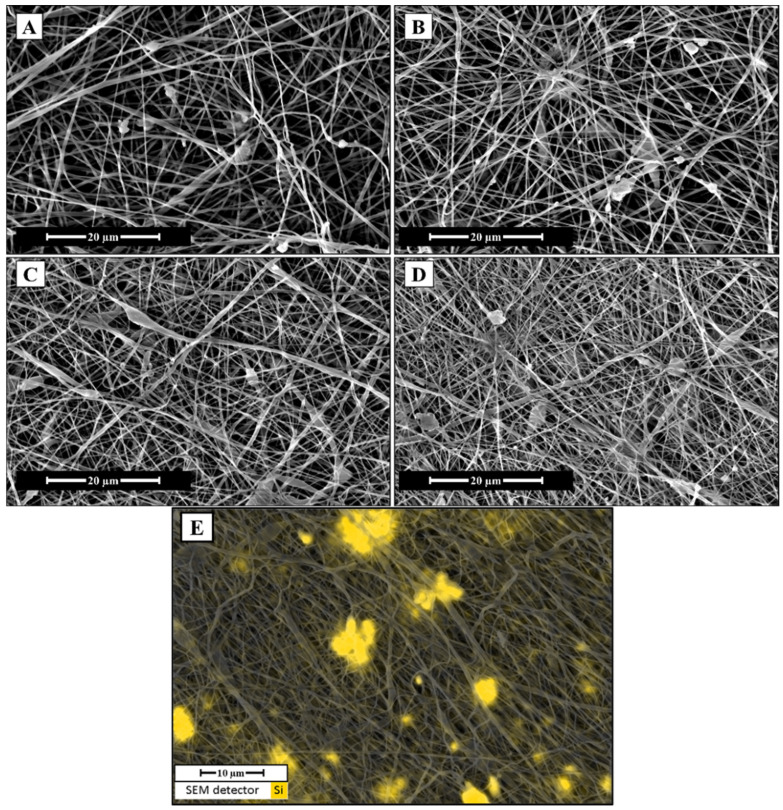
FE-SEM micrographs at 6000× magnification of the as-spun scaffolds: (**A**) 45_9010; (**B**) 45_8020; (**C**) 45_MS_9010; (**D**) 45_MS_8020. (**E**) EDS map of silicon superimposed on a 6000× magnification FE-SEM micrograph of the sample 45_MS_8020.

The structure of the as-spun mats was examined using SEM, and the results are reported in Figure 2. The images revealed the presence of BG particles embedded within the electrospun mats. In all four samples, BG particles were either adhered to the surface of the fibers or completely embedded within them. However, the micrographs in Figure 2 indicate that the BG particles are predominantly incorporated into the fiber structure rather than merely adhered to the surface. Furthermore, the EDS map in Figure 2E, showing the presence of silicon atoms, confirms the widespread distribution of BG within the electrospun mat, further supporting the predominant incorporation of the BG particles into the fiber structure. Although this analysis was performed on all four samples, only the result for sample 45_MS_8020 is shown in Figure 2E for the sake of conciseness. The inclusion of BG particles may have influenced the final morphology of the fibers, as reported in previous studies [43]. Embedded particles occasionally caused bulging along the fibers, which could compromise mechanical performance, as bead-on-string defects are known to introduce weak points in the fiber structure [44,45].

To further evaluate the fiber morphology, an image analysis was carried out using ImageJ software (version 1.54j). The results for the four types of electrospun scaffolds are reported in Table 4. The measured fiber diameters are consistent with previous findings [46,47]. However, a statistical analysis using one-way ANOVA revealed significant differences between the samples (*p* < 0.05). Tukey’s HSD test confirmed that the greatest difference in fiber diameter was observed between 45_MS_9010 and 45_MS_8020. Considering the process parameters listed in Table 3, this difference may be attributed to the variation in applied voltage. The voltage between the needle and the collector has been shown to strongly influence the fiber diameter, with higher voltages generally resulting in thicker fibers compared to lower voltages [37,48]. This effect is likely due to increased Coulombic forces that counteract the viscoelastic resistance of the polymer solution [48]. Nevertheless, the variables affecting fiber diameter are numerous and often difficult to control, including not only setup parameters but also environmental factors such as temperature and humidity [46].

**Figure 3:**
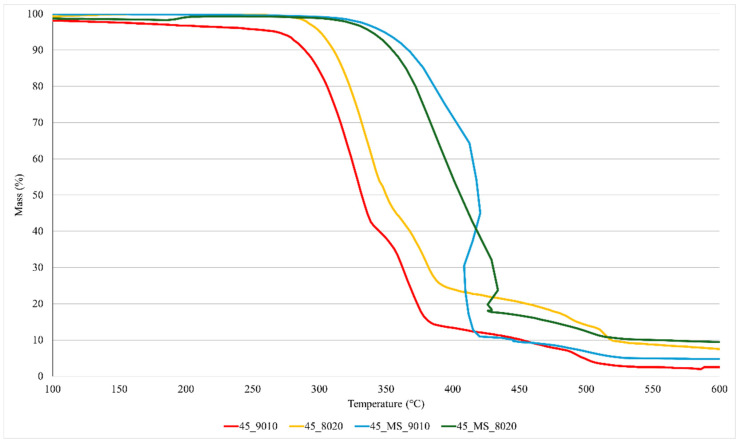
TGA plots showing thermal degradation of PCL and residual BG content in the electrospun mats.

Further evaluation of the BG content in the electrospun mats was performed via TGA, which can provide insights into the thermal stability of the scaffolds, as well as the relative proportions of organic and inorganic components. The TGA plots, reported in Figure 3, show that the degradation of 45S5-loaded PCL began around 300–330 °C. A lower onset temperature of degradation is consistent with the literature findings for electrospun mats incorporating BG, as interactions between the BG particles and the polymer matrix can lead to partial chain scission and decreased thermal stability [49]. Interestingly, the degradation onset for 45S5_MS-based mats occurred at higher temperatures, suggesting a less pronounced interaction with PCL.

As expected, the samples prepared with a PCL-to-BG ratio of 90/10 exhibited a lower BG content, ranging from 3 wt.% to 5 wt.%, while the samples with an 80/20 ratio showed higher BG contents of 8 wt.% to 10 wt.%. Considering the thermal stability of BGs at the temperatures used in TGA (up to 600 °C [9]), the discrepancy between the theoretical BG content and the actual values obtained via TGA may, in the first instance, be attributed to some critical issues in the electrospinning process. In particular, it is likely that solvent–powder interactions and suboptimal jet parameters hinder effective BG transport to the collector, while particle sedimentation—exacerbated by agglomeration—further reduces the incorporation efficiency. The combination of these effects could lead to a theoretical-to-nominal BG incorporation ratio lower than 50%, as observed in this study and reported elsewhere in the literature. For example, recent works have already highlighted similar efficiency issues in the fabrication of BG-loaded electrospun mats [50,51]. In this regard, the suboptimal nominal BG/PCL ratios, compared to the theoretical values of 10 wt.% and 20 wt.%, indicate room for improvement both in the preparation of the suspension for electrospinning and in the optimization of the process parameters. Despite the TGA results, we have chosen to retain the sample labels “9010” and “8020”, referring to the weight ratios used in preparing the electrospinning suspensions. Obviously, these labels were selected for the sake of clarity and consistency but are not intended to imply a direct correspondence to the final BG content in the electrospun scaffolds.

### 3.2. Mechanical Characterization and Surface Wettability

In order to test the physical properties of the electrospun scaffolds, both tensile testing and water contact angle measurements were performed on the samples. Table 5 presents the results of the tensile testing. The results include σ_MAX_ and E in both the L and T directions, expressed in MPa. Five replicates were tested for each direction of each sample and then averaged. Additionally, the results include the mean value of all the measurements taken for each sample, regardless of orientation.

All values of tensile strength in the L direction exceeded 2 MPa, and the Young’s modulus values were all above 4.8 MPa. However, sample 45_MS_9010 showed the highest values, reaching 7.5 MPa and 14.68 MPa, respectively. A two-way ANOVA revealed that, both for the tensile strength and Young’s Modulus, there was low variability within each direction of each sample. However, a statistically significant difference was found between the L and T directions, suggesting anisotropy in the samples. In addition, 45_MS_9010 exhibited significantly higher tensile strength and Young’s modulus in both directions compared to the other samples. The higher mechanical properties of sample 45_MS_9010 could be linked to the lower fiber diameter, which has been reported to heavily influence both the tensile strength and Young’s modulus [52]. Nonetheless, these results appear consistent with the previous literature on the topic [53]. However, the mechanical properties could be further improved, reaching higher tensile strength and Young’s modulus values, as reported for pure PCL electrospun scaffolds by Baudequin et al. [22], even though the presence of embedded glass–ceramic particles may worsen the mechanical properties compared to pure electrospun scaffolds [52]. Further evidence of this behavior can be found when cross-referencing the mechanical properties of the other samples with their respective fiber diameter value. This makes it unlikely that the mechanical properties of the electrospun PCL/BG samples were influenced by the composition of the BG in this work.

Measuring the tensile strength in both the longitudinal and transversal directions revealed the anisotropic nature of the samples. The results show that both the ultimate tensile strength and Young’s modulus are higher in the L-direction compared to the T-direction, indicating an uneven distribution of mechanical properties, likely due to a predominant fiber orientation within the scaffolds. The factors contributing to these phenomena include the type of collector (rotational or stationary), solution composition, voltage, working distance, and flow rate [1,54], all of which can induce anisotropy in mechanical properties. As mentioned in the Materials and Methods section, a rotational collector operating at 300 rpm was employed (which is a relatively low speed compared to other studies aiming to obtain aligned fibers [55,56]). This approach aimed to achieve a homogeneous fiber distribution while minimizing fiber alignment.

To evaluate the surface wettability of the PLC/BG scaffolds, water contact angle measurements were conducted on the samples using double-distilled water. Table 6 summarizes the results along with standard deviations (expressed in degrees °). Each value represents the average of ten measurements taken per sample surface.

The results show that all samples exhibited contact angles above 119°, indicating a hydrophobic surface. Slight differences in contact angle values were observed across the samples, with sample 45_MS_8020 displaying the highest average contact angle. However, a statistical analysis indicated no significant differences in contact angle among the samples (F = 0.565 and *p* = 0.642), suggesting that the wettability of the samples is comparable and that variation in BG composition did not affect surface wettability. This could be related to previous results presented in the micrographs in Figure 2A–D, which revealed that the BG particles are predominantly embedded within the fiber structure. These values fall within the range reported in other studies on similar electrospun scaffolds, which often describe hydrophobicity as a typical feature of electrospun PCL scaffolds [57,58]. To reduce hydrophobicity, surface modification techniques such as plasma treatment or chemical functionalization could be applied to enhance scaffold hydrophilicity, promoting better cell attachment and facilitating tissue integration [59]. 

### 3.3. Tests in Simulated Body Fluid

**Figure 4:**
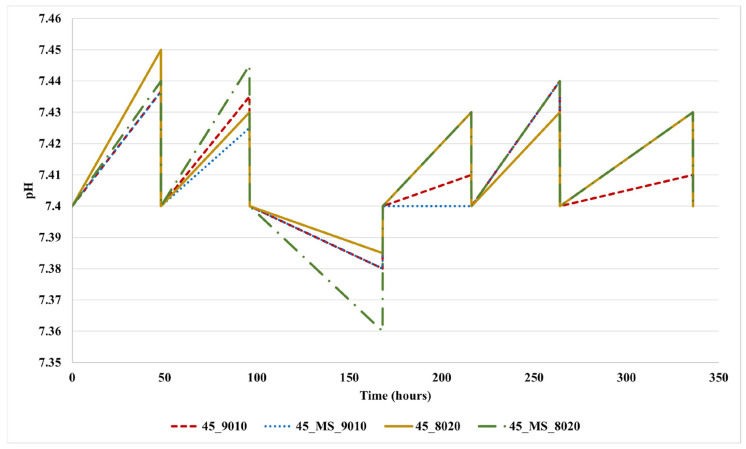
pH variation plot of the 4 electrospun scaffolds, submerged in SBF for 14 days. pH values were recorded every 48 h.

To evaluate the bioactivity of the electrospun PLC/BG scaffolds, SBF testing was conducted according to Kokubo’s protocol. pH changes were monitored in the SBF solution over a 14-day period, with measurements recorded every 48 h. The results are presented in Figure 4. After each measurement, the solution was refreshed to ensure consistent testing conditions and to better mimic the dynamic environment of physiological fluids.

The electrospun samples induced minimal fluctuations in the pH of the solutions, which did not exceed 7.45. This may indicate limited ion exchange and PCL degradation in the solution. The latter hypothesis could be supported by the sudden acidification of the solution measured after the 7th day of immersion. Previous studies have also reported this drop in the pH of the SBF, which could be associated with PCL degradation [60].

**Figure 5:**
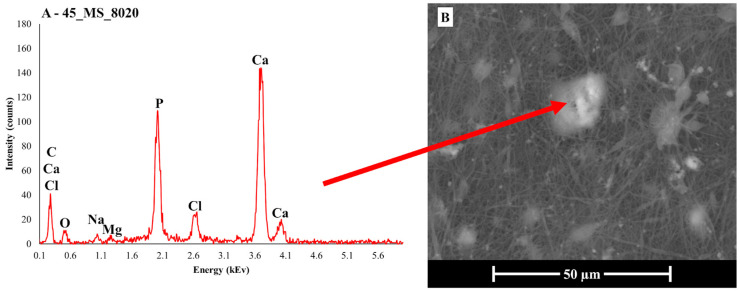
(**A**) EDS analysis of the surface of the 45_MS_8020 electrospun PCL/BG sample and (**B**) SEM image of the analyzed area. The red arrow points to the spot where the analysis was conducted.

Previous research has already established that both 45S5 and 45S5_MS are bioactive, as they precipitated HA after 7 days of immersion in SBF [9,24,25]. Additionally, 45S5_MS demonstrated good biological behavior, exhibiting optimal cell viability and adhesion when tested with human dermal fibroblasts [9]. For these reasons, our investigation focused on EDS analysis of the SBF-treated samples to determine whether calcium- and phosphorus-rich compounds were deposited on the surfaces of the BG particles embedded within the electrospun scaffolds. Figure 5 presents the results of the EDS analysis conducted on sample 45_MS_8020, taken as an example, along with SEM imaging of the sample surface. The analysis revealed a significant concentration of calcium and phosphorus on a white globule, along with the absence of silicon. This could be a potential indicator of HA deposition on the surface of the BG granule in the scaffold structure [61].

Furthermore, the presence of Na and Cl, as detected by the EDS, could be attributed to salt precipitation from the solution [62,63]. Lastly, the SEM image in Figure 5B shows a damaged fiber structure, as the PCL began to degrade upon contact with SBF, as suggested by the acidification phenomenon previously mentioned.

### 3.4. Biological Assessment

#### 3.4.1. In Vitro Evaluation of Cytotoxicity

**Figure 6:**
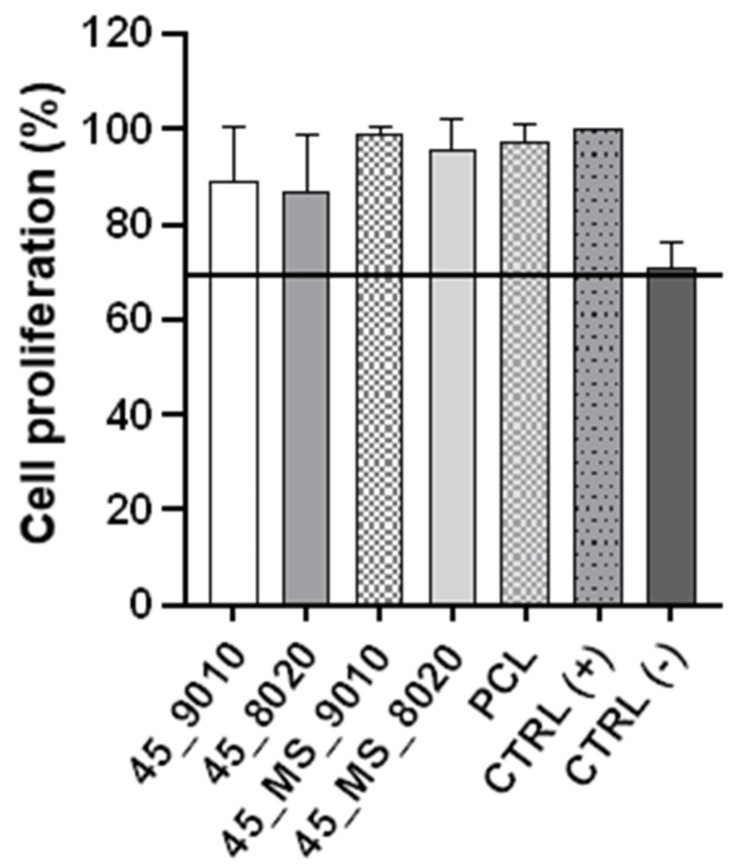
Indirect cytotoxicity test: Proliferation of L929 fibroblasts cultured in electrospun mats extracts, latex extracts, or complete DMEM medium for 24 h. CTRL (+) is the complete DMEM medium, and CTRL (−) is the latex-extract medium. The black line corresponds to 70% proliferation. Statistical analysis was performed using one-way ANOVA with Tukey post-test, with *p* < 0.05 (*n* = 3).

In tissue engineering, 45S5 and PCL are biomaterials that are widely employed. However, the addition of Mg and Sr to the BG composition could lead to an undesirable response in biocompatibility, inducing a decrease in cell viability. To evaluate the cytotoxicity of the mats, an indirect test was conducted according to ISO 10993-5 guidelines. Figure 6 shows the proliferation of the L929 cells that were cultured for 24 h in the extract of electrospun mats and latex and then normalized against the CTRL (+). It is possible to notice that the mats did not demonstrate a significant adverse effect on the cells after culture. For CTRL (−), the proliferation decreased to 70%, which could be related to the cytotoxicity of the latex.

#### 3.4.2. In Vitro Evaluation of Osteogenic Induction Performance

To evaluate whether 45S5 and 45S5_MS play a role in osteogenesis, ALP, an earlier phenotype marker of osteoblast differentiation, and OCN, the most abundant non-collagenous bone protein produced exclusively by osteoblasts, were investigated. In this study, PCL electrospun scaffolds (fiber diameter around 480 nm) served as a control to compare the results obtained with the scaffolds containing BG and additives.

Briefly, bone formation by osteoblasts is divided into two steps: organic matrix formation/deposition and mineralization. Collagen proteins, including type I collagen, are secreted during organic matrix formation, followed by the production of several distinctive non-collagenous proteins, such as ALP, OCN, and osteopontin (OPN). Finally, the calcification process of the extracellular matrix is induced [64]. During matrix mineralization, the secretion of osteocalcin by osteoblasts is associated with the late phase of cell differentiation [65].

**Figure 7:**
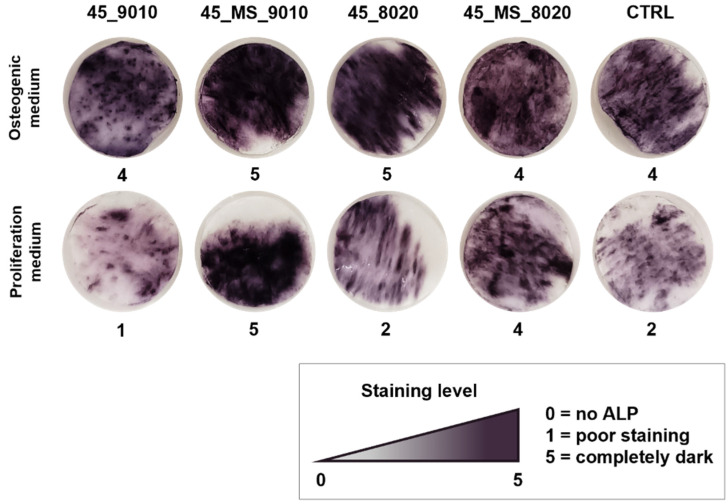
ALP staining of ASC52telo cultured on electrospun scaffolds in proliferation and osteogenic medium after a period of 28 days. A semi-quantitative score was attributed to each sample, from 0 = no ALP presence to 5 = completely dark.

After 28 days of culture, the ALP staining (Figure 7) for samples in the osteogenic medium was more intense than in the proliferation medium, as expected, since the presence of osteogenic factors stimulates cell differentiation.

Stiffness and high surface area are crucial factors to consider when developing an ideal scaffold. Scaffolds designed with biophysical properties that mimic either cancellous or cortical bone provide a proper environment for cells to attach, proliferate, and migrate. Moreover, the interaction between mechanical properties and cell signaling can drive cells to differentiate into a tissue with mechanical properties comparable to the scaffold by influencing cell focal adhesion and the cytoskeleton [66,67]. Furthermore, studies have demonstrated that the osteogenic potential of several stem cells, such as adipose stem cells, is significantly increased by a microfibrous environment [68].

By analyzing the results obtained by the scaffolds in a proliferation medium without osteogenic supplements, it can be inferred that the scaffolds with the highest level of ALP staining were those loaded with 45S5_MS, as well as the reference scaffold made of pure PCL. Specifically, PCL and 45_MS_9010 showed the highest E and the lowest fiber diameter, which are favorable properties for cell differentiation. Interestingly, in the case of sample 45_MS_8020, which had a lower Young’s Modulus value (5.37 MPa) and a larger fiber diameter (1.62 µm), it also showed a high level of ALP staining. A previous study demonstrated that pure PCL and composite PCL electrospun scaffolds could induce bone differentiation without an osteogenic medium, and this differentiation could be more related to the morphology (alignment, multiple layers) and composition of the material than to the relationship between fiber diameter and stiffness [22]. It could be hypothesized that, in this study, the increase in ALP staining may be linked more closely to the presence of the doping ions in the BG (i.e., magnesium and strontium) than to the size and/or mechanical properties of the fibers.

**Figure 8:**
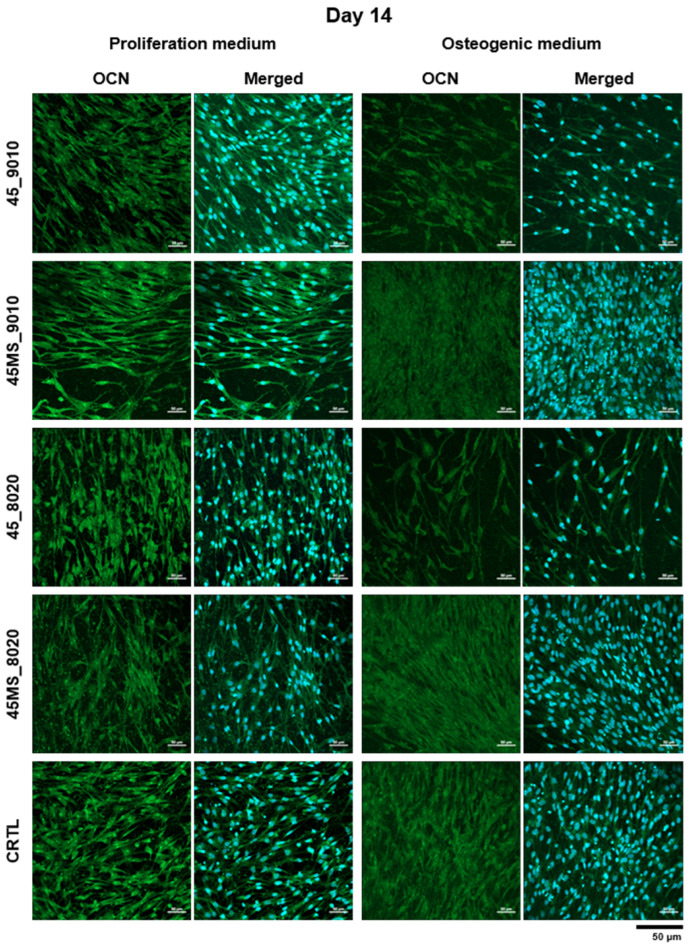
Immunofluorescence staining of osteocalcin (green) and cell nuclei (blue) in ASC52telo cultured on electrospun scaffolds in two media (proliferation and osteogenic) on day 14. Scales bars represent 50 µm (magnification 20×).

**Figure 9:**
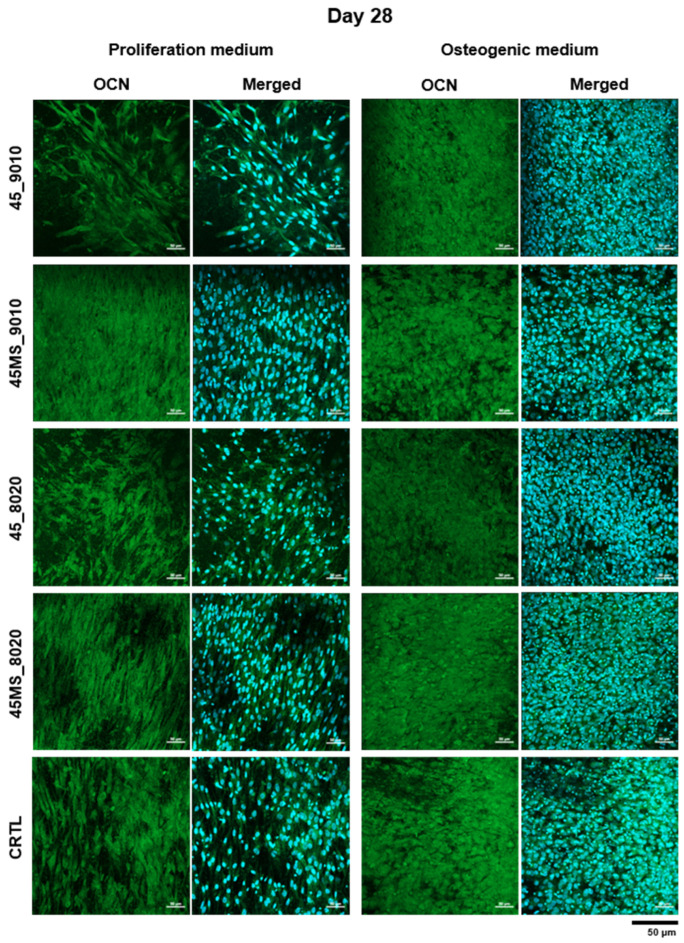
Immunofluorescence staining of osteocalcin (green) and cell nuclei (blue) in ASC52telo cultured on electrospun scaffolds in two media (proliferation and osteogenic) on day 28. Scales bars represent 50 µm (magnification 20×).

The fluorescence images in Figure 8 and Figure 9 show stained nuclei (blue) and osteocalcin (green) in the ASC52telo after 14 and 28 days of cell culture. On day 14, it was possible to observe the presence of OCN expression in all samples in both the proliferation and differentiation media, with enhanced OCN presence in the osteogenic medium. From day 14 to 28, an increase in OCN expression was observed for all samples, highlighting that the cells continued their growth process and suggesting differentiation towards osteoblasts.

Despite the slightly hydrophobic characteristics of the electrospun samples, which could compromise cell adhesion, these results demonstrate that ASC52telo can adhere, spread, and proliferate on the scaffolds, indicating that the inclusion of 45S5 and 45S5_MS can provide a favorable microenvironment for cell growth.

Furthermore, following the ALP staining results, on day 14, a higher cell density was observed in the mats containing Mg and Sr. Several studies have indicated the advantages of bio-ceramics doped with magnesium and strontium, which can promote bone formation [12,69,70,71]. The presence of Mg can stimulate the proliferation of osteoblasts [72], whereas Sr possesses therapeutic effects on osteoporosis by enhancing pre-osteoblastic differentiation [73]. In vivo studies have shown that BG doped with strontium and BG doped with magnesium increase new bone regeneration by stimulating osteoblast metabolic activity and proliferation [74,75]. Another study revealed that the presence of Mg in tricalcium phosphate ceramic stabilized the cell–material interface and promoted higher cell attachment and growth [69,72,74].

These results demonstrate that the inclusion of enriched BG into PCL electrospun scaffolds might provide a favorable environment for cells and represent an alternative for tissue engineering.

## 4. Conclusions

Electrospun composite mats were produced using PCL and incorporating melt-quenched BG powders into the structure of the scaffolds. The mats underwent extensive testing, including mechanical characterization, surface wettability assessment, SBF testing, and biological evaluation. The BG powders were successfully incorporated into the electrospun mats. This was achieved thanks to a thorough grinding of the particles, which reduced their size to 10^−6^–10^−7^ m, allowing the particles to be small enough to be successfully electrospun and embedded into the scaffold structure.

The results can be summarized as follows. The mechanical characteristics of the mats were consistent with previous research on PCL and composite electrospun mats, indicating a minimal influence of the BG on the structural integrity of the scaffolds. The surface wettability was low, reflecting the high hydrophobicity of the scaffolds. This outcome is attributed to the hydrophobic nature of PCL, combined with the microfibrous morphology of the mats. The analysis of the samples after SBF testing suggests that the BG was accessible to the solution and capable of forming apatite-like crystals. However, other precipitates containing high concentrations of Na^+^ and Cl^−^ ions were also observed, as confirmed by EDS analysis. Furthermore, the solution could induce degradation of PCL, which caused the acidification of the immersion medium. 

Indirect cytotoxicity tests with L929 mice cells confirmed the non-cytotoxicity of the electrospun scaffolds, with viabilities in the range of 85% and 100%. ALP activity testing revealed that the presence of Mg and Sr was associated with higher ALP staining, which could be favorable for cell differentiation. Fluorescence imaging revealed that, from days 14 to 28, osteocalcin expression persisted, suggesting cell proliferation and differentiation, especially in the scaffolds with additives.

These results suggest that the inclusion of Mg- and Sr-enriched BG powders, produced via the traditional melt–quench technique, can serve as a viable alternative to unloaded PCL electrospun scaffolds. Not only do they enhance biological performance, but they also preserve the key characteristics of pure PCL. Further work should focus on optimizing the processing parameters for scaffold fabrication and improving the surface wettability, as this could enhance the scaffold’s healing properties by promoting better cell adhesion to the fibers. Additional research could also explore other properties of BGs, such as their angiogenetic potential, which supports vascularization and wound healing, and their antibacterial activity, which helps reduce infection risk. These properties could have a significant impact on the treatment of soft tissue wounds and patient outcomes, potentially opening new applications for composite electrospun BG/PCL scaffolds.

## Figures and Tables

**Table 1:** Compositions of 45S5 [7] and 45S5_MS [9].

Oxide	45S5 Composition (mol%)	45S5_MS Composition (mol%)
SiO_2_	46.1	46.1
CaO	26.9	26.9
Na_2_O	24.4	9.4
P_2_O_5_	2.6	2.6
MgO	-	5
SrO	-	10

**Table 2:** Compositions of the precursor suspensions (expressed in percentages), with the relative BG and PCL content used before electrospinning.

Sample	BG	PCL Beads (%)	BG Powder (%)
45_9010	45S5	90	10
45_8020		80	20
45_MS_9010	45S5_MS	90	10
45_MS_8020		80	20

**Table 3:** Electrospinning parameters used for spinning the samples in this study. The table includes the environmental parameters, which were measured at the beginning of the electrospinning process.

Sample	Voltage (kV)	Flow Rate (mL/h)	Working Distance (cm)	Total Spun (mL)	Ambient Parameters ^1^	
					Ambient Temp. (°C)	Relative Humidity (%)
45_9010	18	1.5	15	3.3	22.1	68.3
45_8020	18	1.5	15	3.3	22.0	68.3
45_MS_9010	17.8	1.5	15	3.3	21.7	70.2
45_MS_8020	20	1.5	15	3.3	21.8	71.2

^1^ Ambient parameters were measured in the laboratory at the start of the electrospinning process and are dependent on weather conditions.

**Table 4:** Results of the image analysis: mean fiber diameter, mean BG particle size, and the percentage of the area occupied by particles in the images.

Sample	Mean Fiber Diameter (µm)	Mean BG Particle Size (µm^2^)	Area Occupied by Particles (%)
45_9010	1.58 ± 0.32	2.57	1.16
45_8020	1.03 ± 0.23	2.44	1.85
45_MS_9010	0.53 ± 0.16	1.41	1.09
45_MS_8020	1.62 ± 0.46	1.73	1.99
PCL ^1^	0.5–1	-	-

^1^ Data for PCL, added as reference, was obtained from Baudequin et al. [22].

**Table 5:** Ultimate tensile strength and Young’s Modulus of the samples measured via tensile testing. Two-way ANOVA showed significant statistical differences among samples and directions (*p* < 0.05) for both mechanical properties.

Sample	Orientation	σ_MAX_ (MPa)	Young’s Modulus (MPa)
45_9010	L	2.21 ± 0.37	4.84 ± 0.62
	T	1.94 ± 0.33	4.43 ± 0.92
45_8020	L	4.32 ± 0.52	6.37 ± 1.64
	T	3.14 ± 0.57	5.58 ± 1.02
45_MS_9010	L	7.48 ± 0.48	14.68 ± 1.61
	T	4.79 ± 0.52	8.74 ± 0.68
45_MS_8020	L	2.16 ± 0.24	5.37 ± 0.43
	T	1.37 ± 0.14	4.49 ± 1.15
PCL ^1^	L	-	14.89

^1^ Data for PCL, added as reference, were obtained from Baudequin et al. [22].

**Table 6:** Contact angle values of the five electrospun mats with standard deviation. One-way ANOVA showed no significant differences among the samples (F = 0.565, *p* = 0.6416).

Sample	Angle Mean Value (°)
45_9010	119.68 ± 2.52
45_8020	119.82 ± 1.63
45_MS_9010	120.04 ± 2.80
45_MS_8020	120.94 ± 2.40
PCL ^1^	122.8 ± 5

^1^ Data for PCL, added as reference, were obtained from Baudequin et al. [22].

## Data Availability

The original contributions presented in this study are included in the article. Further inquiries can be directed to the corresponding author.

## Abbreviations

The following abbreviations are used in this manuscript:

BG	Bioactive Glass
PCL	Poly (ε-caprolactone)
SBF	Simulated Body Fluid
HA	Hydroxyapatite
ALP	Alkaline Phosphatase
OCN	Osteocalcin
OPN	Osteopontin
DCM	Dichloromethane
DMF	Dimethylformamide
DMEM	Dulbecco’s Modified Eagle Medium
PBS	Phosphate-Buffered Saline
DPBS	Dulbecco’s Phosphate-Buffered Saline
FBS	Fetal Bovine Serum
(E)SEM	(Environmental) Scanning Electron Microscope
CTRL ±	Control Positive/Negative
σ_MAX_	Ultimate Tensile Strength
E	Young’s Modulus
L/T	Longitudinal/Transversal
EtOH	Ethyl Alcohol
